# Editorial: Phage display: Technique and applications

**DOI:** 10.3389/fmicb.2022.1097661

**Published:** 2022-12-06

**Authors:** Jian Huang, Yoichi Takakusagi, Beibei Ru

**Affiliations:** ^1^School of Life Science and Technology, University of Electronic Science and Technology of China, Chengdu, Sichuan, China; ^2^Institute for Quantum Life Science, National Institutes for Quantum Science and Technology, Chiba, Japan; ^3^Cancer Data Science Lab, Center for Cancer Research, National Cancer Institute, National Institutes of Health, Bethesda, MD, United States

**Keywords:** phage display, next generation sequencing, artificial intelligence (AI), peptide, antibody, phage library

Phages are the most abundant organisms on the earth and have a great impact on the earth's ecosystems. Some phages have also been engineered into good expression vectors for coupling genotypes and phenotypes, which can be utilized to harness the power of evolution by artificial selection. Phage display is a powerful and versatile technology for constructing a library of peptides or antibodies displayed on phage virions, and then screening them for peptides or antibodies with desired properties. In 2018, one-half of the Nobel Prize in chemistry was awarded jointly to George P. Smith and Sir Gregory P. Winter for the “phage display of peptides and antibodies” (Smith, 2019; Winter, 2019). Phage display has been widely used to map epitopes, identify drug targets (Takakusagi et al., 2020), and develop therapeutics, diagnostics, and vaccines.

In the present Research Topic, we have organized 11 articles, including seven original research papers, two method papers, and two reviews, to share the authors' understanding of the phage display technique and its applications from various angles.

Next-generation sequencing (NGS) has been employed in most, if not all, fields of biology and medicine. Phage display is no exception, either for antibodies or peptides. Using the Roche 454 NGS platform, Lisowska et al. identified over 13,000 peptides targeting ubiquitin and over 10,000 peptides against ubiquitin-like modifier NEDD8 from the Ph.D.-12 Phage Display Peptide Library. Two peptides were further confirmed to inhibit both E3 ubiquitin ligases, MDM2 and CHIP, and their binding modes were solved by the NMR analysis. Zhao et al. made a phage panning scheme that separately covered selections against phosphotyrosine (pTyr) and sulfated tyrosine (sTyr) peptides, followed by NGS. After mining the NGS data and validating with experiments, they successfully identified some sTyr superbinders. Maruthachalam et al. designed three synthetic Fab libraries based on a modified trastuzumab framework. Using the Ion Torrent NGS platform, they found 12 Fabs against Notch-1, and 2 Fabs showed strict specificity for Notch-1 with very high affinities.

Artificial intelligence (AI) is also increasingly applied in phage display studies. He et al. used NGS with phage display and a lot of peptides were obtained from the Ph.D.-12 Phage Display Peptide Library panning against PD-L1. Based on the NGS data obtained, they further adopted different sequence features and various machine-learning methods to train models for predicting PD-L1 binders. Finally, PDL1Binder, an ensemble computational model, was implemented as a web server. Liu et al. studied the sequence features of phage proteins and non-phage proteins. They proposed a feature selection method based on ensemble learning. Their findings might help to find out new phages from metagenomes, providing candidate genes or vectors for new phage library construction.

In addition to NGS and AI, relevant scholars have been trying to improve the phage display technique and explore its application from different aspects. In the review by André et al., the state-of-the-art *in vivo* phage display methodologies were summarized and discussed, especially the promising emerging selection strategy for improving antibody targeting and drug delivery properties. Allen et al. reviewed how bioconjugation and the incorporation of non-canonical amino acids had expanded the chemical diversity of peptides and proteins displayed by M13 phage virions for a variety of purposes. Ma et al. developed and compared three different methods to increase the signal-to-background ratio of ELISA assay during biopanning, which might help probe the weak protein–protein interactions using phage display. Thavorasak et al. applied phage display to reveal the enhancing epitope on the spike protein of the porcine epidemic diarrhea virus (PEDV), which would be helpful and useful in designing a safe and effective PEDV vaccine devoid of the enhancing epitope. Glab-ampai et al. tested the effect of a phage display-derived superantibody originally targeting a conformational epitope on RNA-dependent RNA polymerase of the hepatitis C virus. Their results showed that the superantibody could also inhibit the replication of many RNA viruses, such as DENV, ZIKV, JEV, EV71, CVA16, PEDV, and SARS-CoV-2 (Wuhan wild-type and the variants of concern) in a dose-dependent manner. Chen et al. developed a method called Pi-mqPCR, which was short for phage display mediated immuno-multiplex quantitative PCR. They applied it to monitor and distinguish the differences in the immune response to antigenic domains of multiple SARS-CoV-2 variants simultaneously.

In conclusion, the phage display technique is very flexible and evolves continuously. It has been applied more and more frequently with NGS and AI, which makes phage display more productive and efficient. In our opinion, phage display is still one of the most useful experimental techniques, which greatly helps biomarker discovery, vaccine design, and peptide and antibody drug development (Ning et al., 2021).

## Author contributions

All authors listed have made a substantial, direct, and intellectual contribution to the work and approved it for publication.
